# Supplementary material for: Predicting visual function by interpreting a neuronal wiring diagram
Source: Nature. 2024 Oct 2;634(8032):113–23. doi: 10.1038/s41586-024-07953-5 (PMC11446822; doi:10.1038/s41586-024-07953-5)
Supplement: Supplementary file 6 — CRF and ERF predictions for individual TmY4 and TmY9 cells. Analogous to Supplementary Data 3, but for TmY target types. Shown are the top four monosynaptic pathways, the strongest pathway passing through each of the top ten intermediary types (ranking from Extended Data Fig. 7), and the trisynaptic pathway Tm1–TmY–Dm3–TmY (see the section entitled Prediction of spatial normalization). [file 41586_2024_7953_MOESM6_ESM.zip › DataS4/TmY9qΓèÑ/720575940636888553.pdf]

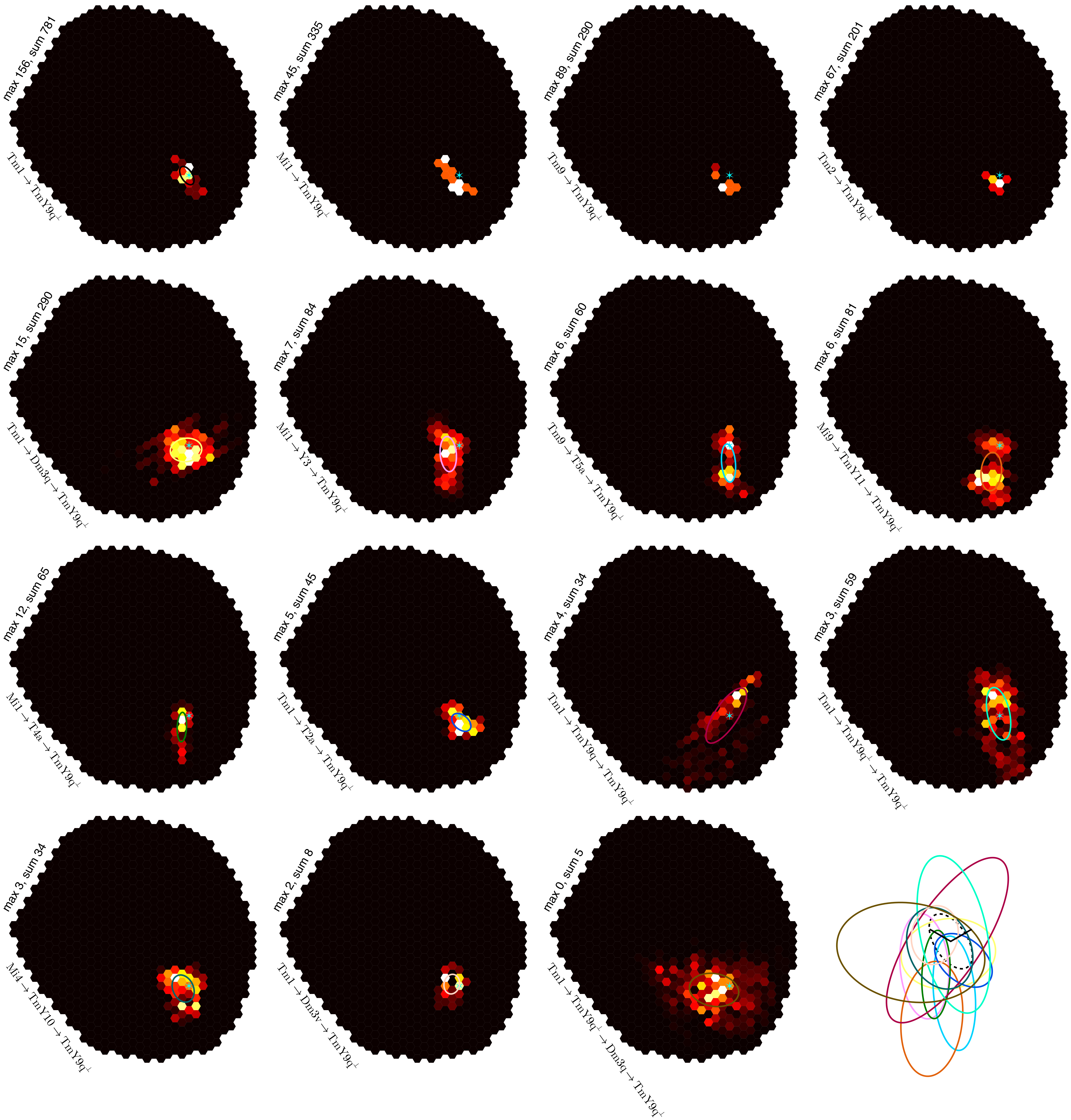

max 156, sum 781
Tm1 → TmY9q⊥
max 45, sum 335
Mi1 → TmY9q⊥
max 89, sum 290
Tm9 → TmY9q⊥
max 67, sum 201
Tm2 → TmY9q⊥
max 15, sum 290
Tm1 → Dm3q → TmY9q⊥
max 7, sum 84
Mi1 → Y3 → TmY9q⊥
max 6, sum 60
Tm9 → T5a → TmY9q⊥
max 6, sum 81
Mi9 → TmY11 → TmY9q⊥
max 12, sum 65
Mi1 → T4a → TmY9q⊥
max 5, sum 45
Tm1 → T2a → TmY9q⊥
max 4, sum 34
Tm1 → TmY9q⊥ → TmY9q⊥
max 3, sum 59
Tm1 → TmY9q⊥ → TmY9q⊥
max 3, sum 34
Mi4 → TmY10 → TmY9q⊥
max 2, sum 8
Tm1 → Dm3v → TmY9q⊥
max 0, sum 5
Tm1 → TmY9q⊥ → Dm3q → TmY9q⊥
